# AGO2 slicing of a domesticated retrotransposon is necessary for normal vasculature development

**DOI:** 10.1101/2025.04.02.646793

**Published:** 2025-04-03

**Authors:** Manish Kumar, Andrea G. Maria, Mahendra Prajapat, Joana A. Vidigal

## Abstract

Argonaute (AGO) mediated slicing of RNA—also known as RNAi—is a highly conserved phenomenon that is evolutionarily linked to the repression of transposons and other repeats. Although RNAi is no longer a major mechanism of repeat control in mammals, AGO2 has retained cleavage competence and is able to efficiently cut RNAs with extensive complementarity to a bound guide. The regulatory roles this activity plays in mammals however remains poorly understood. Here we show that mice carrying two catalytically inactive *Ago2* alleles have extensive developmental abnormalities including systemic vascular defects that are characterized by enlarged and leaky vessels and stem from endothelial cell dysfunction. Endothelial cell defects are caused by failure to repress *Rtl1* , a paternally-imprinted domesticated retrotransposon, whose cleavage in wild-type animals is triggered by miRNAs of the maternally-imprinted *miR-433∼127* cluster. Our data pinpoint an essential mRNA cleavage target of AGO2 and suggest that the repurposing of a TE-Argonaute regulatory interaction contributes to the retention of AGO catalytic competence in mammals.

### INTRODUCTION

Transposable elements (TEs) are selfish repetitive sequences. They pose an imminent threat to their hosts because of their ability to mobilize to other genomic locations (Bourque et al. 2018; Wells and Feschotte 2020) which can disrupt essential genes or regulatory motifs and thus compromise both the health of tissues (Kazazian and Moran 2017; Rodriguez-Martin et al. 2020; Burns 2022) and the integrity of the germline (Klattenhoff et al. 2007; Toth et al. 2016). In order to protect the genome of their cells from this risk, organisms have evolved many mechanisms to restrain TE activity (Maksakova et al. 2008), including RNA interference (RNAi) (Watanabe et al. 2008; Sala et al. 2023). RNAi is an ancestral RNA-silencing pathway (Fire et al. 1998) thought to have arisen as a rudimentary innate defense system against exogenous virus and endogenous repeats (Obbard et al. 2009). At the center of this pathway is a catalytic Argonaute (AGO), which directly slices target transcripts that have perfect or near-perfect complementarity to a bound guide (Zamore et al. 2000; Becker et al. 2019). This is an efficient strategy to repress retrotransposons, a class of TEs that mobilizes through a 'copy and paste' mechanism via an RNA intermediate (Wicker et al. 2007).

The dangers posed by TE mobilization are balanced by the contributions they provide to their hosts. About 40% of mammalian genomes originate from retrotransposons (Lander et al. 2001; Mouse Genome Sequencing et al. 2002) and although a few of these sequences can still mobilize and thus remain a threat, the vast majority has been inactivated through mutations (Ostertag and Kazazian 2001; Szak et al. 2002). Many of these TE remnants have been repurposed to perform essential host functions (Senft and Macfarlan 2021; Modzelewski et al. 2022). TE domestication has played a major role in genome evolution, with many genes and regulatory sequences that are essential to animal physiology originating from repurposed TE-host interactions (Flemr et al. 2013; Notwell et al. 2015; Chuong et al. 2016; Chuong et al. 2017; Sakashita et al. 2020; Modzelewski et al. 2021).

Similarly, mechanisms that repress TE expression have also been co-opted during evolution. Although repression of repeats through RNA cleavage remains a common function of AGO proteins in many eukaryotes (Vastenhouw and Plasterk 2004; Matzke and Birchler 2005; Chung et al. 2008; Chapman et al. 2022), it is no longer a major role for mammalian Argonautes which have instead been re-purposed for endogenous gene regulation via the miRNA pathway (Bartel 2018; Sala et al. 2020). Repression by miRNAs does not require slicing. Instead, short sequence complementarity between an AGO-bound miRNA and an mRNA's 3'UTR leads to recruitment of a large effector complex that destabilizes the target and represses its translation (Jonas and Izaurralde 2015). Yet AGO2, one of the four Argonaute proteins encoded in mammalian genomes, retains full catalytic competence via the highly conserved DEDH tetrad in its PIWI domain (Liu et al. 2004; Nakanishi et al. 2012). The regulatory functions of this tetrad remain poorly understood in mammals. What is clear is that slicing is physiologically important since mice homozygous for a point mutation that inactivates the tetrad ($Ago2^{D598A/D598A}$) die a few hours after birth (Cheloufi et al. 2010; Jee et al. 2018). The causes behind this lethality are unknown and no gross morphological defects have been identified in these animals, apart from anemia which is caused by a block in the maturation of two atypical miRNAs that require AGO2 slicing for their biogenesis. While severe, anemia is not the cause of animal death (Jee et al. 2018), pointing to the existence of other RNAs whose cleavage by AGO2 is essential. Although a few highly complementary miRNA targets have been identified that can be cleaved by AGO2 (Yekta et al. 2004; Davis et al. 2005; Karginov et al. 2010; Shin et al. 2010; Hansen et al. 2011), the physiological importance of that cleavage is unknown.

To further understand the biological requirements for AGO catalytic competence in mammals, we re-examined litters from $Ago2^{+/D598A}$ intercrosses. We found that homozygous mutants have extensive developmental abnormalities including systemic vascular defects in both extra-embryonic and embryonic tissues. Vascular defects stem from dysfunction of the endothelial cell layer leading to enlarged and leaky vessels. Befitting AGO's ancestral role in TE restriction, we find that these are caused by derepression of *Rtl1*, a retrotransposon-derived paternally-imprinted gene whose cleavage by AGO2 is essential for endothelial cell development and function. Thus, an event of transposon domestication that preserved its regulatory relationship with a catalytic AGO likely contributed to the retention of that endonucleolytic function in mammals.

## RESULTS

### Phenotypic abnormalities in the absence of AGO2 slicing competence

To define the phenotypic requirements for AGO2's catalytic competence in mice we analyzed animals in which this activity is selectively disrupted through a point mutation in the conserved catalytic domain of the protein ($Ago2^{D598A}$) (Cheloufi et al. 2010). As previously reported, homozygous mutants could be recovered at the expected Mendelian ratio at embryonic day (E) 18.5 but died a few hours after birth with evidence of anemia (Cheloufi et al. 2010; Jee et al. 2018). Animal death was accompanied by respiratory distress although neither histological analysis nor µCT scans of whole embryos revealed morphologic abnormalities in the lungs or the heart. As previously observed (Cheloufi et al. 2010), we also found evidence of internal hemorrhages in some embryos (**Figure 1A**).

In addition to these previously characterized defects, we also documented several other developmental phenotypes in the absence of AGO2 slicing competence. First, the majority of E18.5 $Ago2^{D598A/D598A}$ fetuses had omphalocele, an abdominal wall closure defect in which the intestine protruded outside the body cavity but remained inside a membranous sac (**Figure 1B-D**). This defect correlated with the presence of small visceral cavities in these animals and was largely resolved by birth, suggesting a delay in resolving the physiological umbilical hernia which is naturally present in wild-type animals from E12.5-E16.5. Although liver development was normal, all mutant animals analyzed by µCT (n=9) also displayed misshapen liver lobes which could be secondary to their partial extrusion from the visceral cavity (**Figure 1E**, top). Second, µCT scans also revealed that all mutants had abnormally thick abdominal muscles, particularly evident for the transversus thoracic muscle (**Figure 1F**, arrow). An abnormally thick pleuropericardial membrane was also evident in some embryos (**Figure 1F**, asterisk). Alongside these minor defects in cavity membranes and abdominal muscle, we observed major defects in the diaphragms of all mutants. Specifically like the other abdominal structures discussed above, both the diaphragm muscle and the diaphragm cruxes of $Ago2^{D598A/D598A}$ animals were significantly thicker than those of controls (**Figure 1E**, bottom; **Figure 1F**, arrow heads). In all cases the diaphragm muscle also displayed a jagged appearance in contrast to its smooth shape in wild-type animals (**Figure 1E**, bottom; **Figure 1F**, arrow heads). Third, loss of AGO2 catalytic activity led to a dose-dependent increase in placental weights (**Figure 1G, Figure 1A**) with no significant change in the weight of the embryos (**Figure 1H, Sup Figure 1B**), reflecting a significantly reduced placental efficiency in both heterozygous and homozygous mutants (**Figure 1I, Supplementary Figure 1C**). Increased placental weights correlated with increased levels of phosphorylated histone 3 (**Supplementary Figure 1D**) suggesting that placentomegaly is a result of increased cell proliferation in this organ. Finally, although not evident in the weight (**Figure 1H**), all *Ago2*$^{D598A/D598A}$ embryos we analyzed displayed some degree of growth retardation, pronounced spinal curvatures, and shorter bones compared to controls. Particularly, we documented a slight decrease in the sizes of the scapula, humerus, and radius but not of other bones (**Supplementary Figure 2**). Because *Hoxb8* is one of the few known cleavage targets of AGO2 in the developing embryo (Yekta et al. 2004), we also looked for the presence of other patterning abnormalities in catalytic mutants. Despite the importance of HOXB8 for normal vertebral development (van den Akker et al. 1999), the posterior homeotic transformations caused by *Hoxb8* overexpression (Charite et al. 1994), and the conservation of the *miR-196*/*Hoxb8* cleavage interaction across species (Yekta et al. 2004; He et al. 2011), we found no skeletal patterning defects in *Ago2*$^{D598A/D598A}$ animals (**Supplementary Figure 2**).

In summary, we have undertaken the first comprehensive characterization of mice that lack AGO2 catalytic competence. Our data shows that in addition to the well-documented block in erythrocyte development that causes anemia in catalytic mutant mice (Cheloufi et al. 2010; Jee et al. 2018), these animals display numerous phenotypic abnormalities in both embryonic and extra-embryonic tissues, highlighting an extensive impact of AGO2-mediated slicing in mammalian physiology. Which of these defects are directly caused by loss of AGO2 cleavage and which are secondary consequence of dysfunctions in other tissues is not clear.

**Placental defects are epiblast-derived**

The internal hemorrhages combined with the placental defects observed in homozygous mutants suggested that a primary consequence of loss of AGO2 catalytic activity maybe the dysfunction of the vascular system. An axis between placental and cardiovascular development is well-established (Riley et al. 1998; Adams et al. 2000; Raffel et al. 2009; Maruyama et al. 2016) and placental defects are strongly correlated with abnormal vasculature differentiation in both extra-embryonic and embryonic tissues (Perez-Garcia et al. 2018). Deficient vascularization of the placenta by embryonic vessels also compromises the exchange of nutrients, gases, and waste between mother and fetus (Rossant and Cross 2001) which can result in growth restriction (Krishna and Bhalerao 2011) and early lethality (Perez-Garcia et al. 2018) as observed in our mutants.

The placenta is a complex organ, with many cell types involved in establishing and supporting the maternal-fetal interface (Rossant and Cross 2001). Trophectoderm-derived cells generate the majority of placental structures while the epiblast—which gives rise to the embryo proper and is characterized by *Sox2* expression—contributes to vascularization of the placenta by giving rise to the endothelial cells that line the fetal-derived vessels of that organ (**Supplementary Figure 1G**) (Rossant and Cross 2001). To determine if the placental defects arise from loss of catalytic activity in the epiblast-derived cells that give rise to its network of embryonic vessels, we crossed *Ago2*$^{+/D598A}$*; Sox2-Cre*$^{+/-}$ males to *Ago2*$^{flx/flx}$ females. This leads to epiblast-specific recombination of the floxed allele in embryos that carry the *Cre* transgene, generating animals that express a single *Ago2* allele in cells from that lineage, which is either catalytic incompetent (*Ago2*$^{flx/D598A}$*;Sox2-Cre*$^{+/-}$) or wild-type (*Ago2*$^{flx/+}$*;Sox2-Cre*$^{+/-}$) (**Supplementary Figure 1H**). As expected, animals expressing only a catalytic incompetent AGO2 recapitulated all major developmental defects in the fetus including failure to close the abdominal wall resulting in a persistent hernia at E18.5 (**Figure 2A**, **2B**). These animals also died prematurely, with no *Ago2*$^{flx/D598A}$*;Sox2-Cre*$^{+/-}$ animals present at weaning (**Figure 2C**). Importantly, just like *Ago2*$^{D598A/D598A}$ animals, *Ago2*$^{flx/D598A}$*;Sox2-Cre*$^{+/-}$ mutants had significantly heavier placentas at embryonic day E18.5 as well as lower placental efficiency compared to controls (**Figure 2D-F**). These data indicate that the placental overgrowth we have observed is not derived from extraembryonic tissues but rather originates from a dysfunction in epiblast-derived cells. This dysfunction seems to culminate in the increased proliferation of non-endothelial cells in the placenta (**Supplementary Figure 1E-1F**), which drives the overgrowth of that organ in the absence of AGO2 catalytic competence. Since epiblast-derived cells in the placenta differentiate into the endothelium that lines the fetal vessels of that organ (Rossant and Cross 2001), these data further suggest that AGO2 catalytic competence maybe essential for proper vessel formation during mammalian embryogenesis.

**AGO2 slicing activity is essential for vessel development**

To gain additional insights into the requirement for AGO2 slicing we performed RNA sequencing of *Ago2*$^{+/+}$ and *Ago2*$^{D598A/D598A}$ placentas. We found numerous dysregulated genes in homozygous mutants (**Figure 3A**). In line with a putative role for AGO2 catalytic activity in vascular development there was a significantly enrichment of gene ontology terms related to this process amongst the genes upregulated in our *Ago2*$^{D598A/D598A}$ placentas (**Figure 3B**, left). Notably, upregulated genes were also associated with fully penetrant neonatal lethality in addition to phenotypes related to vascular dysfunction (**Figure 3B**, right). To understand which cell types are affected by loss of AGO2 catalytic competence, we looked for enrichment of gene signatures of every placental cell type—defined by single-cell RNA sequencing (Marsh and Blelloch 2020)—amongst upregulated genes. This highlighted a significant enrichment for vascular and lymphatic endothelial gene signatures in our dataset further supporting the notion that loss of AGO2 catalytic competence specifically affects endothelial cell development (**Figure 3C**, **Supplementary Fig 3A**).

We looked at vasculature development by performing immunofluorescence against CD31, an endothelial cell marker. We found that both *Ago2*$^{D598A/D598A}$ and *Ago2*$^{flx/D598A}$*;Sox2-Cre*$^{+/-}$ animals had abnormally patterned fetal-derived vessels, which were significantly enlarged in the placenta and yolk-sacs of mutants compared to controls (**Figure 3D**, **Supplementary Figure 3B-3E**). These vascular defects extended to the fetus as well, which at E18.5 also showed evidence of vessel dilation (**Figure 3E**) including in the heart (**Figure 3F**). Moreover, histological analysis by a trained pathologist blind to the genotypes identified a generalized edema—i.e. tissue swelling caused by uncontrolled fluid extravasation—in some of these embryos which was most prominent in the subcutaneous tissue. This suggests dysfunction of the endothelial barrier function in the absence of AGO2 catalytic competence resulting in vessels with higher permeability. Extreme barrier disruption in some animals may also underlie the internal hemorrhages that we (**Figure 1A**) and others (Cheloufi et al. 2010) have observed in some animals at birth.

**Abnormal miRNA processing is not the cause of gene dysregulation in mutant placentas**

Loss of AGO2 catalytic activity can impact the maturation of miRNAs (Cheloufi et al. 2010; Cifuentes et al. 2010; Yang et al. 2010; Jee et al. 2018; Kotagama et al. 2024; Pal et al. 2024), with dysregulation of *miR-451* and *miR-486* resulting in a block in erythrocyte development that leads to anemia in $Ago2^{D598A/D598A}$ animals (Cheloufi et al. 2010; Jee et al. 2018). To determine if abnormal miRNA processing might also underlie the vasculature defects in these animals, we sequenced small RNAs in $Ago2^{D598A/D598A}$ and littermate $Ago2^{+/+}$ placentas. As expected, *miR-451* was significantly downregulated in homozygous mutant samples, whereas *miR-144* which is expressed from the same transcript remained unchanged (**Figure 4A**). This reflects the *Ago2* catalysis-dependent biogenesis of the former. A few other miRNAs showed differences in expression between the two datasets, totaling 12 dysregulated miRNAs (9 upregulated, 3 downregulated; **Figure 4A**, **Supplementary Table 1**). All these miRNAs arise from characteristically structured hairpin precursors suggesting they mature through a typical biogenesis pathway that does not require AGO2 slicing.

To determine if changes in miRNA abundance could nevertheless result in the transcriptomic dysregulation observed in our mutants, we looked for evidence that predicted targets of these miRNAs were preferentially impacted in $Ago2^{D598A/D598A}$ placental samples. We found no support for that claim (**Figure 4B-C**), suggesting that the majority of the miRNA expression changes are indirect consequences of loss of AGO2 cleavage activity.

***Rtl1*, a domesticated transposon is a cleavage target of AGO2**

Alternatively, we hypothesized that some placental miRNAs could direct AGO2 to cleave transcripts whose consequent upregulation in mutants would cause the vascular defects we describe here. To test that hypothesis, we computationally searched amongst upregulated transcripts for the presence of sequences with perfect complementarity to miRNAs with at least 30 reads per million (RPM) in our dataset (**Supplementary Table 2**). A single transcript stood out from this analysis, *Rtl1* (**Figure 5A**), which was both significantly upregulated in our mutants (**Figure 5B**) and had 10 perfectly complementary sites to well-expressed miRNAs (**Figure 5A**, **Supplementary Table 3**).

*Rtl1* (Retrotransposon-like protein 1; also known as *Peg11*, paternally expressed gene 11) is an imprinted domesticated transposon that is expressed solely from the paternal chromosome (**Figure 5C**) (Charlier et al. 2001; Cavaille et al. 2002). The maternal chromosome expresses instead an imprinted noncoding RNA that overlaps the *Rtl1* locus and serves as the primary transcript for a cluster of 6 miRNAs (*miR-136*, *miR-432*, *miR-434*, *miR-127*, *miR-433*, *miR-431*) (**Figure 5C**) (Seitz et al. 2003), five of which are expressed above the 30 RPM threshold in mouse placentas (*miR-136*, *miR-434*, *miR-127*, *miR-433*, *miR-431*). Because these miRNAs are transcribed in an antisense manner relative to *Rtl1*, their sequences have perfect complementarity to that gene (**Figure 5D**, **Supplementary Table 3**) and can trigger AGO2-mediated cleavage of *Rtl1* at the position opposite to nucleotides 10 and 11 in the small RNA sequence (Davis et al. 2005). We looked for evidence of such cleavage products in total RNA extracted from wild-type placentas using a modified 5'RACE protocol (**Figure 5E**, **Supplementary Figure 4A-4B**) (Kobayashi et al. 2010). We were able to detect such products for all five well-expressed miRNAs from this cluster, and in two instances for passenger strands as well, for a total of 7 confirmed cleavage sites on the *Rtl1* transcript. These cleavage products were, as expected, absent from RNAs extracted from $Ago2^{D598A/D598A}$ placentas indicating that they are a direct result of AGO2's slicing activity (**Figure 5E**). Importantly, sequencing of these 5'RACE amplicons confirmed that—in the large majority of cases—the 5'adaptor had ligated to a 5'phosphate group at the predicted cleavage position within the target sequence (**Figure 5D; Supplementary Figure 4B**), further confirming that these are true physiological AGO2 cleavage events.

#### *Rtl1* upregulation causes endothelial defects in *Ago2*$^{D598A/D598A}$ animals

While loss of RTL1 results in clogged fetal capillaries and concomitant growth retardation of both fetus and placenta, its overexpression causes enlarged fetal vessels and placentomegaly as seen in our mutants (Sekita et al. 2008). Although previous work concluded that *Rtl1* functions are placental-specific (Sekita et al. 2008), we detected upregulation of *Rtl1* not only in the placentas of *Ago2*$^{D598A/D598A}$ (**Supplementary Figure 5A**) and *Ago2*$^{flx/D598A}$*;Sox2-Cre*$^{+/-}$ mice (**Supplementary Figure 5B**), but also in the fetus itself (**Supplementary Figure 5A**), particularly in isolated CD31$^{+}$ endothelial cells (**Figure 6A-6C**). This is in line with the widespread vascular defects we documented in the absence of AGO2 cleavage activity.

The similarities between the phenotypes observed in the absence of AGO2 catalytic competence and *Rtl1* overexpression suggest that de-repression of this transcript when cleavage by AGO2 is disrupted is responsible for the endothelial defects we documented. To test if that is the case, we established endothelial cell cultures from *Ago2*$^{D598A/D598A}$ animals and *Ago2*$^{+/D598A}$ controls (**Supplementary Figure 5C**). These cultures recapitulated the *Rtl1* upregulation (**Figure 6D**) as well as every vascular phenotype we observed in homozygous mutants. These included decreased proliferation (**Supplementary Figure 5C-5D**), increased permeability (**Figure 6E**, **Supplementary Figure 5E**), and abnormal tube formation (**Figure 6F-5I**) resulting in meshes with significantly enlarged lumens (**Supplementary Figure 5F**) that mirrored the enlarged vessels we observed in both embryos and placentas. Thus, endothelial cell culture faithfully recapitulates phenotypes observed *in vivo*, allowing for further genetic manipulation to determine the contribution of *Rtl1* to these defects.

To test whether *Rtl1* upregulation is responsible for the vascular defects we have observed in *Ago2* catalytic mutants, we sought to restore *Rtl1* repression in *Ago2*$^{D598A/D598A}$ cells in an RNAi-independent manner. To do so, we stably infected both lines with a vector expressing a fusion between a nuclease-inactive Cas9 (dCas9) and the Krüppel-associated box (KRAB) repressor, which allows for the epigenetic repression of targeted loci (Thakore et al. 2015). Using this vector, we targeted the dCas9-KRAB fusion to the promoter region of *Rtl1* using anpreviously validated gRNA (Sanson et al. 2018). Unlike *Ago2*$^{D598A/D598A}$ cells infected with a control guide, those that received a guide against *Rtl1* showed levels of that transcript statistically indistinguishable from those of controls (**Figure 6D**). Remarkably, this single gene manipulation was sufficient to rescue all phenotypes of *Ago2*$^{D598A/D598A}$ mutant endothelial cells including proliferation (**Supplementary Figure 5C-5D**), permeability (**Figure 6E**, **Supplementary Figure 5E**), and tube formation (**Figure 5F-5I**, **Supplementary Figure 6F**). Together, these data show that the vascular defects we observed in *Ago2*$^{D598A/D598A}$ mice are caused by the failure to cleave *Rtl1* transcripts whose consequent upregulation causes the widespread defects in the endothelium of these animals.

### DISCUSSION

Befitting Argonaute's ancestral role in restricting endogenous repeats, we found that *Ago2* catalytic mutant animals have widespread vascular defects caused by failure to cleave a domesticated retrotransposon. It is noteworthy that these stem from derepression of the paternally-imprinted *Rtl1* gene, given that imprinting is thought to have evolved as a mechanism to balance gene dosage (Ishida and Moore 2013). Thus, *Rtl1* expression—like that of other imprinted genes—may need to be kept within a narrow window to be compatible with mammalian physiology. In the case of *Rtl1*, this is dose sensitivity seems to be managed not only through imprinting, but also through direct cleavage of its mRNA by AGO2. Our works extends the known impact of loss of AGO2's slicing activity in mammals, which aside from causing erythropoietic defects due to a block in maturation of two noncanonical miRNAs (Cheloufi et al. 2010; Jee et al. 2018) also causes endothelial cell dysfunction due to a failure to cleave the *Rtl1* transcript. Both *Rtl1* and the miRNAs that drive its cleavage are conserved in placental mammals (Edwards et al. 2008; Kaneko-Ishino and Ishino 2012; Kozomara and Griffiths-Jones 2014). As a result, we expect that this slicing interaction has conserved roles in endothelial cells across eutherians, representing an additional physiological requirement for slicing that likely contributed to the retention of a catalytic competent Argonaute.

In addition to a dysfunction of the endothelial cell layer, *Ago2*$^{D598A/D598A}$ animals have extensive abdominal defects including omphalocele and abnormal skeletal muscle development. Given the respiratory distress of catalytic mutant animals at birth, it is possible that these abnormalities— in particular those that affect the diaphragm—contribute to the neonatal lethality of the mutants. These defects too are likely a direct consequence of *Rtl1* upregulation in the absence of cleavage, as mice that overexpress *Rtl1* have severe abnormalities in abdominal, intercostal, and diaphragm muscles that resemble those in our mutants (Kitazawa et al. 2020). While no abdominal hernia has been reported in those animals, omphalocele is a prominent feature of individuals with Kagami-Ogata syndrome (KOS; Omim #608149), a disorder caused by genetic disruptions at the 14q32 imprinted locus where *RTL1* is located that result in the overexpression of paternally imprinted protein-coding genes (*DLK1*, *RTL1*, *DIO3*) and loss of numerous maternally imprinted non-coding RNAs (Kagami et al. 2005; Ogata and Kagami 2016). Several genes in this region have been previously implicated in muscle development and suspected to play a role in the etiology of KOS (Waddell et al. 2010; Gao et al. 2015; Prasasya et al. 2020; Dill et al. 2021), but upregulation of *RTL1* is amongst the most likely molecular events driving the disease (Kagami et al. 2008; Sekita et al. 2008; Kitazawa et al. 2020). The resemblance between the phenotypes of AGO2 catalytic mutants and the features of KOS (**Supplementary Table 4**)—which also include placentomegaly and respiratory distress (Prasasya et al. 2020)—lends support to this model. Thus, disruption of AGO2-mediated cleavage, which in KOS patients is achieved through the loss of the *miR-136~miR-431* miRNA cluster, seems to severely impact human health as well, further highlighting the importance of Argonaute slicing in mammals.

It is not clear if de-repression of *Rtl1* is sufficient to explain the neonatal lethality of $Ago2^{D598A/D598A}$ animals. Mice that inherit a targeted deletion of the *Rtl1* locus from their fathers ($Rtl1^{+/\Delta Pat}$) or from their mothers ($Rtl1^{+/\Delta Mat}$) and hence lose the expression of either *Rtl1* or the miRNAs that target it, die perinatally with developmental abnormalities related to the ones we describe here (Sekita et al. 2008; Kitazawa et al. 2020). This shows that both loss and overproduction of *Rtl1* impacts viability. While $Rtl1^{+/\Delta Pat}$ animals die at mid-to-late gestation (E15.5-E19.0), $Rtl1^{+/\Delta Mat}$ mice—which are functionally identical to $Ago2^{D598A/D598A}$ in terms of *Rtl1* upregulation (Davis et al. 2005)—survive to birth but die within a day, essentially phenocopying the lethality of *Ago2* catalytic mutants (Sekita et al. 2008; Cheloufi et al. 2010). Yet, the impact of *Rtl1* dysregulation on animal viability is highly dependent on genetic background, as both $Rtl1^{+/\Delta Pat}$ and $Rtl1^{+/\Delta Mat}$ animals survive in a mixed C57BL/6 x 129/Sv background but die when backcrossed to C57BL/6 for 6-10 generations (Sekita et al. 2008; Kitazawa et al. 2020). This does not seem to be the case for the $Ago2^{D598A}$ strain which was originally generated in a 29S6/SvEvTac x C57BL/6Ncr background before being backcrossed to C57BL/6, with no reported changes in phenotype penetrance (Cheloufi et al. 2010). The discrepancy between background dependency in $Rtl1^{\Delta}$ and $Ago2^{D598A}$ strains suggests that, while upregulation of *Rtl1* likely contributes to the lethality of *Ago2* catalytic mutants, other yet unknown cleavage targets of AGO2 may also play a role (Sala et al. 2023).

About 25% of people diagnosed with Kagami-Ogata Syndrome also die in early childhood. Respiratory problems are the major cause of death in these patients, who often also suffer from cardiovascular malformations (Kagami et al. 2015; Ogata and Kagami 2016). In addition, they are at risk of developing cancer, particularly hepatoblastoma which has an incidence of 5-10% in these patients (Kagami et al. 2015; Ogata and Kagami 2016). This too may be a consequence of *Rtl1* upregulation since expression of this domesticated retrotransposon has been shown to drive tumor initiation in the liver (Riordan et al. 2013), promote the proliferation of cancer cells (Fan et al. 2017), and correlate with poor prognostic features (Mahmoudi et al. 2021). As such, it is possible that cleavage of *Rtl1* by AGO2 is also important to prevent cell transformation, and that disruption of this slicing is a critical event driving tumorigenesis in some malignancies.

### ACKNOWLEDGMENTS

We thank all members of the Vidigal lab and of LBMB for discussions and comments on this work. We thank Pedro Rocha, Alex Kelly, Michael Lichten, Sarah Sheppard, Katie McJunkin, Karl Pfeifer, and Todd Macfarlan for critical review of this manuscript. We thank the NCI's Laboratory Animal Sciences Program, in particular D. Gallardo and M. Figueroa, for expert mouse care and help maintaining the mouse colonies. We also thank the NCI's molecular histopathology core, especially B. Karim and the Mutant Mouse Resource & Research Center (MMRRC) at UC Davis, especially Louise Lanoue for invaluable pathology work. This work utilized the computational resources of the NIH HPC Biowulf cluster (hpc.nih.gov). This work was supported by the Intramural Research Program of the National Institutes of Health through the Center for Cancer Research, National Cancer Institute, project 1ZIABC011810 (J.A.V.).

### AUTHOR CONTRIBUTIONS

MK and JAV conceived the project. MK performed the experiments and analyzed the data with help from MP, AGM, and JAV. JAV supervised the project. JAV wrote the manuscript with input from all authors.

### COMPETING INTERESTS

The authors declare no competing interests.

## METHODS

### Mouse husbandry and lines.

All animal procedures were conducted according to the NIH Guide for the Care and Use of Laboratory Animals, under Animal Study Proposal no. 390923 approved by the Animal Care and Use Committee of the National Cancer Institute. *Ago2*$^{D598A}$ mice—carrying a point mutation that converts the aspartic acid at position 598 to alanine inactivating AGO2's catalytic tetrad—(Cheloufi et al. 2010), *Ago2*$^{flx}$ mice—carrying loxP sites around exons 9-11 of the Ago2 gene—(O'Carroll et al. 2007), and Sox2-Cre—which express Cre recombinase under the control of the Sox2 promoter—(Hayashi et al. 2002) were purchased from the Jackson laboratory (Stock nos. 014151, 016520, and 008454 respectively). Weaned mice were genotyped using DNA extracted from tail clippings. Embryos were genotyped using DNA extracted from yolk-sacs.

### µCT analysis.

µCT analysis was performed at the Mutant Mouse Resource & Research Center (MMRRC) at UC Davis. A total of 2 *Ago2*$^{+/+}$ and 9 *Ago2*$^{D598A/D598A}$ littermate animals were processed and analyzed by a trained pathologist. Both genotypes were compared to data from C57BL/6N wild-type mice.

### Immunofluorescence.

Tissues were collected and fixed in 10% neutral buffered formalin overnight at room temperature and embedded in paraffin. 5-µm thick tissue sections were dewaxed in xylene and rehydrated through an ethanol series. Sodium citrate buffer [10mM sodium citrate (Millipore Sigma #130-097-418) was used for antigen retrieval, and sections were blocked in blocking buffer [(3% FBS (VWR #97068-091), 1% BSA (Millipore Sigma #A9647), 0.3% Triton X-100 (Millipore Sigma #T8787) in PBS)] prior to incubation with primary antibodies overnight at 4°C. Sections were washed (0.3% Triton X-100 in PBS) and a fluorescence-conjugated secondary antibodies were added for 1 h. After washing, sections were counterstained with DAPI. Sections were mounted with ProLong Gold Antifade reagent. Images were collected using a Axioscan slide scanner (Zeiss Axioscan 7) and analyzed using HALO image analysis platform.

**Flow cytometry analysis.**

*Ago2*$^{+/+}$ and *Ago2*$^{D598A/D598A}$ placentas were isolated from timed pregnancies of *Ago2*$^{D598A/+}$ intercrosses at E16.5. Tissues were resected and briefly washed in cold PBS, before being minced and enzymatically digested [(2.5mg/mL collagenase A (Millipore Sigma #10103586001) 1U/mL Dispase II (Millipore Sigma #4942078001)] in 1X Hank's Balanced Salt Solution (HBSS). The cell suspension was then filtered through a 70 µm cell strainer (Corning #431751) followed by 40 µm cell strainer (Corning #431750) and pelleted at 400g for 5 minutes at 4°C. Cells were resuspended in 200 µl cold FACS buffer (3% FBS/1%BSA in PBS) and Fc receptors blocked with anti-CD16/32 clone 2.4G2 antibody (BD Pharmingen #553141) for 10 min on ice. Endothelial cells were labelled using the anti-CD31 antibody (BD Bioscience #740879) for 30 min on ice in the dark. Cells were washed twice with FACS buffer and centrifuged at 220g for 5 min at 4°C. Cell pellets were fixed with 4% PFA (Electron Microscopy Sciences #15714) diluted in PBS for 15 min at room temperature. Cells were further washed with FACS buffer and permeabilized with permeabilization solution (0.3% Triton X-100 in FACS buffer) for 10 min at room temperature. Cells were centrifuged at 220g for 5 min at 4°C and resuspended in 200 µl antibody dilution buffer (0.1% Triton X-100 in FACS buffer) and labelled with anti-Ki67 antibody (Invitrogen #25569880) for 40 min on ice. Cells were washed twice in FACS buffer and resuspended in 400 µl FACS buffer. Finally, 50 µl of CountBright Absolute Counting Beads (Thermo Fisher Scientific #C36950) were added into falcon round bottom tubes with cell strainer cap (BD Falcon #352235) directly prior to analysis on a Sony ID7000 FACS machine. Spectral data was processed using the FlowJo software.

**Detection and sequencing of AGO2 cleavage products.**

Total RNA was extracted from E18.5 placentas using TRIzol (Thermo Fisher Scientific #15596018). Genomic DNA was removed with TURBO DNA-free kit (Thermo Fisher Scientific #AM1907). For each sample, polyadenylated RNA was isolated from 100 µg of total RNA using Dynabeads mRNA purification kit (Thermo Fisher Scientific #610-06) following manufacturer's instructions. The purified mRNA was used to ligate 5' RNA adaptor (100 μM) by mixing T4 RNA ligase buffer (NEB # B0216), 10 mM ATP (NEB # P0756), RNaseOUT (Termo Fisher Scientific #10777-019), and T4 RNA ligase (NEB # M0204) as previously described (Li et al. 2019). A second round of mRNA purification was performed to remove unincorporated 5' RNA adaptors. Adaptor-ligated mRNA was reverse-transcribed using SuperScript™ II (TermoFisher, Cat. No. 18064) by combining transcript-specific primers and a β-actin-specific primer. cDNA was amplified with a forward primer designed to bind to the 5' RNA adaptor sequence and a gene-specific reverse primer using the high-fidelity Platinum Taq DNA Polymerase (Thermo Fisher Scientific #11304011) supplemented with DMSO (Thermo Fisher Scientific #F-515) and betaine (Thermo Fisher Scientific #J77507.VCR). The PCR cycling conditions were as follows: 94°C for 2 min, 35 cycles of 94°C for 20 s, 60°C for 30 s, and 68°C for 45 s; and 72°C for 7 min.

PCR products were run on 2% agarose gel and extracted using Zymoclean Gel DNA recovery kit (Zymo #D4008) following manufacturer's instruction. Extracted amplicons were cloned using Zero Blunt TOPO PCR cloning kit (Thermo Fisher Scientific #450245), and plasmid DNA isolated from individual colonies sent for sanger sequencing.

**Isolation and culture of placenta endothelial cells.**

Endothelial cells were isolated from E16.5 placentas. Tissues were resected and briefly washed in cold PBS, before being minced and enzymatically digested [(2.5mg/mL collagenase A (Millipore Sigma #10103586001) and 1U/mL Dispase II (Millipore Sigma #4942078001)] in Hank's Balanced Salt Solution (HBSS). The cell suspension was then filtered twice through cell strainers (70 μm followed by 40 μm) and pelleted at 400g for 5 minutes at 4°C. The cell pellets were resuspended in 100 μl cold FACS buffer (1X PBS, 0.5% BSA, 2mM EDTA) and used to purify endothelial cells using an initial lineage cell depletion step (Miltenyi Biotec #130-090-858) followed by endothelial cell purification using anti-CD31-conjugated magnetic beads (mouse CD31, Miltenyi Biotec #130-097-418) according to the manufacturer's protocol. $CD31^+$ cells were seeded on Fibronectin (Millipore Sigma #F0895) coated 48-well plate (Thermo Fisher Scientific #657165) and cultured in EGM-2MV microvascular endothelial cell growth medium (Lonza-

CC-3202). The purity of isolated primary endothelial cells was confirmed by immunostaining using CD31 antibody (Cell Signaling 77699).

**Generation of stable cell lines.**

Pure primary cultures of endothelial cells from *Ago2*$^{D598A/+}$ and *Ago2*$^{D598A/D598A}$ mice were immortalized through infection with a lentivirus expressing a myristoylated-Akt1 (kind gift from Dr. Jason Butler, Hackensack Meridian Health, USA) as previously described (Kobayashi et al. 2010). To knockdown *Rtl1*, cells were transduced with either a control dCas9-KRAB-T2a-GFP lentivirus (Addgene #71237) or a lentivirus expressing a gRNA against *Rtl1* (ACCAAGTCTCGCTCTCGGGC). The knockdown of Rtl1 was confirmed through qRT-PCR.

**Quantitative RT-PCR.**

RNA was isolated using TRIzol reagent (Thermo Fisher Scientific #15596018) and DNase treated with TURBO DNA-free kit (Thermo Fisher Scientific #AM1907) following manufacturers' protocols. RNA was reverse transcribed with SuperScript IV (Thermo Fisher Scientific #18090200) using Oligo-dT primers. Relative expression level of *Rtl1* was quantified using Sybr Green (Thermo Fisher Scientific #4309155) and normalized to either *Beta-actin* or *Gapdh* housekeeping gene. Primer sequences are as follows: *Rtl1* (GCTCAGAGGCAATCAAGGAG, CGGTTGGAAAGGAGTGTGC), *Beta-actin* (TGGAGCCACCGATCCACACA, CGTTGACATCCGTAAAGACC), *Gapdh* (CGTCCCGTAGACAAAATGGT, TCAATGAAGGGGTCGTTGAT).

**Western blot.**

Tissues were homogenized on ice in 500 µl SDS-lysis buffer (2% SDS, 50mM Tris, 10% Glycerol) containing protease inhibitors (Millipore Sigma #PPC2020) and protein concentration was determined using the Pierce BCA protein assay kit (ThermoScientific #23225). Samples were stored at -80$^{0}$ C until analysis. Protein extracts were mixed with Laemmli sample buffer (Bio-Rad #1610747), boiled for 5 minutes, and chilled briefly on ice prior to loading on gels. Proteins were resolved by NuPAGE™ Bis-Tris

Mini Protein Gels, 4–12% (Thermo Fisher Scientific #NP0322BOX) electrophoresis and transferred to nitrocellulose membrane (Thermo Fisher Scientific #88018) by electroblotting. Membranes were blocked in 5% BSA (Millipore Sigma #A9647) in Tris-buffered saline (10 mM Tris, pH 7·5, 150 mM sodium chloride) containing 0.1% Tween 20 (TBST). The p-Histone H3 (Santacruz #sc-8656; dilution 1:500), Cleaved-caspase-3 (Cell Signalling #9661; dilution 1:500), Caspase-3 (Cell Signalling #9662; dilution 1:500), Histone H3 (Abcam #ab1791; dilution 1:2000), and Gapdh (Santacruz #sc-32233; dilution 1:4000) antibodies were used in TBST, 5% BSA overnight at 4°C. Membrane were washed and anti-Rabbit IgG HRP (Cell Signaling #7074) or anti-mouse IgG HRP (Cell Signaling #7076) were added for 1 h. Proteins were developed using Supersignal West Dura Extended duration substrate (Thermo Fisher Scientific #34076) and visualized by chemiluminescence (Amersham ImageQuant 800 systems).

**Skeletal Preparations.**

Embryos (E18.5) were eviscerated and soaked in dd$H_2O$ for 3 h at room temperature (17-22°C) as described previously (de Pontual et al. 2011). Fetuses were heat shocked in a 65°C water bath for 30 s to facilitate removal of skin. Embryos were fixed in absolute ethanol (Millipore Sigma #E7023) followed by acetone (Millipore Sigma #179124) overnight at room temperature. Cartilages were soaked in alcian blue (Millipore Sigma #A5268) solution (150 mg/l alcian blue 8GX, 80% ethanol, 20% acetic acid) and alizarin red (Millipore Sigma #A5533) solution (50 mg/l alizarin red S in 2% KOH) to stain for cartilage and bone respectively. Remaining tissues were cleared in 2% KOH (Millipore Sigma #105033) dissolved in dd$H_2O$, and skeletons were placed in 25% glycerol (Millipore Sigma #G5516) for storage. Images were captured with a Zeiss Stereo Discovery V8 microscope and processed in Photoshop. Measurements of bone length were performed in ImageJ.

**Permeability assay**

Permeability of endothelial cells assayed using the *in vitro* Vascular Permeability Assay kit (Millipore Sigma #ECM642) following manufactures' instructions. Briefly, cells were cultured on 96-well transwell inserts until confluency. Cells were cultured in either endothelial cell growth medium or endothelial cell growth medium supplemented with 20ng/ml VEGF (Peprotech #450-32) for another 24 h. FITC-conjugated dextran was added to the upper compartment of the inserts. One hour after adding dextran, the fluorescence intensity of the medium in the lower compartment was measured by the fluorescent micro plate reader (Bio Tek Synergy Multimode reader) at an excitation of 485 nm and emission of 538 nm.

**Tube formation assay**

Endothelial cells were plated on Matrigel basement membrane matrix (Corning #354234) coated 48-well culture plate (Thermo Fisher Scientific #152640) at a density of 10,0000 cells per well. Endothelial tube formation was monitored for up to 12h, at which point cells were imaged. Images were analyzed using ImageJ software for total number of junctions, meshes, nodes, and total mesh area.

**Total RNA sequencing**

RNA was isolated from E18.5 $Ago2^{+/+}$ and $Ago2^{D598A/D598A}$ placentas using TRIzol reagent (Thermo Fisher Scientific #15596018) and treated with TURBO DNA-free kit (Thermo Fisher Scientific #AM1907). Ribosomal RNA was depleted using the Ribo-Zero Plus kit (cat# 20040525). Libraries were constructed using the Illumina TruSeq Stranded Total RNA Library. Sequencing was performed on NextSeq 2000 with PE100 sequencing. Reads were trimmed using cutadapt (v 4.7) and mapped to GRCm39 genome using STAR aligner (2.7.11b). Read alignments were counted using Subread featureCounts (v 2.0.3). Differentially expressed genes were identified with DEseq2 (R 3.6.0).

**Small RNA sequencing**

Libraries were constructed using NEBNext® Multiplex Small RNA Library Prep Set for Illumina® (cat #E7300S) following manufacturer's instructions. Sequencing was performed on NextSeq 500 using SR75. Reads were trimmed using cutadapt (v 4.7). Trimmed reads were mapped to either miRNA or hairpin customized genomes using STAR aligner, with sequences retrieved from miRBase (2.7.11b). Read alignments were counted using Subread featureCounts (v 2.0.3) where features were retrieved from miRBase. Differentially expressed mature and precursor miRNAs were identified using DEseq2 (R 3.6.0).

**DATA availability**

FASTQ and processed files RNA sequencing datasets produced in this study can be found at the Gene Expression Omnibus (GEO) (GSE291207 and GSE291208).

### FIGURE LEGENDS

**Fig. 1. Extensive developmental defects in the absence of AGO2 cleavage competence. (A)** Hematoxylin-Eosin-stained sagittal sections showing examples of abdominal hemorrhages (arrowhead) in *Ago2* catalytic mutants. **(B)** Gross appearance of wild-type (+/+) and catalytic mutant (D598A/D598A) animals at E18.5. Int., intestine; U.C., umbilical cord. **(C)** Hematoxylin-Eosin-stained sagittal section of omphalocele. **(D)** Penetrance of omphalocele in E18.5 animals. **(E)** Micro-CT sagittal sections of wild-type (+/+) and catalytic mutant (D598A/D598A) animals at E18.5. Arrows point to liver deformation in mutants; arrowheads highlight the diaphragm. **(F)** Micro-CT transverse sections of wild-type (+/+) and catalytic mutant (D598A/D598A) animals at E18.5. Note the thickened transversus thoracic muscle (arrow) and pleuropericardial membrane (asterisk). Arrowheads highlight diaphragm muscle and cruxes which are also significantly thickened and misshapen compared to controls. Labels: 1, right and left crux of diaphragm muscle; 2, left lobe of liver; 3, left middle lobe of liver; 4, Falciform ligament, 5, diaphragm; 6, diaphragm. **(G)** Relative placental weights of mutants relative to wild-type littermates at 3 developmental stages. **(H)** Relative embryo weights of mutants relative to wild-type littermates at 3 developmental stages. **(I)** Relative placental efficiency of mutants relative to wild-type littermates at 3 developmental stages. Each dot represents an animal. p-values were calculated using a two-tailed t-test.

**Fig. 2. Characterization of animals with epiblast-specific loss of AGO2 catalytic competence. (A)** Gross appearance of (flx/+) and (flx/D598A) Sox-2+ animals at E18.5. Int., intestine; U.C., umbilical cord. **(B)** Penetrance of omphalocele in E18.5 animals for all genotypes. **(C)** Observed and expected numbers of genotypes recovered at weaning (P30). p-value was calculated using the chi-squared test. **(D)** Relative placental weights of mutants relative to littermate controls at E18.5. **(E)** Relative embryo weights of mutants relative to littermate controls at E18.5. **(F)** Relative placental efficiency of mutants relative to littermate controls at E18.5. Each dot represents an animal. p-values were calculated using a two-tailed t-test.

**Fig. 3. Loss of AGO2 catalytic competence causes wide-spread vascular defects. (A)** Volcano plot showing genes significantly dysregulated in *Ago2* mutant placentas (FDR<-0.05 and [$\log_2$(fold-change)] > 1.5). **(B)** Enriched GO terms (left) and MGI Phenotypes (right) amongst upregulated genes. Terms related to vascular development are highlighted in red. Neonatal lethality is highlighted in pink. **(C)** Gene Set Enrichment Analysis (GSEA) plots showing a significant enrichment of gene signatures for vascular and lymphatic endothelium amongst the genes dysregulated in *Ago2* catalytic mutants. **(D)** Left, immunofluorescence staining of mouse placentas with an antibody against the CD31 endothelial cell marker. Right, quantification of vessel area in wild-type and mutant placentas. p-values we calculated using a tow-tailed t-test. **(E)** Hematoxylin-Eosin-stained sagittal sections of E18.5 embryos, showing enlarged vessels in mutant (bottom, closed arrow heads) compared to wild-type (top, open arrow heads) animals. **(F)** Quantification of vessel area in wild-type and mutant hearts.

**Fig. 4. Transcriptomic changes are not caused by miRNA dysregulation. (A)** Analysis of small RNA libraries in wild-type and mutant placentas. We consider well-expressed miRNAs those with more that 30 RPM in these datasets. The biogenesis of *miR-486* and *miR-451* was previously shown to depend on AGO2 slicing activity, and both of are highlighted in the plot along with *miR-144* which is co-transcribed with *miR-451*. **(B)** Cumulative fraction plots showing that the expression of predicted targets of dysregulated miRNAs is not preferentially impacted. **(C)** As in (B) but highlighting the targets of individual downregulated (blue tones) or upregulated (red tones) miRNAs by number of predicted target sites.

**Fig. 5. *Rtl1* is a cleavage target of AGO2 during embryogenesis. (A)** Volcano plot of genes significantly upregulated genes in *Ago2* mutant placentas (FDR<-0.05 and $\log_2$(fold-change) > 1.5 labeled by presence (dot color) and number (dot size) of perfect binding sites for well-expressed miRNAs. **(B)** *Rtl1* expression in mouse placentas, as measured by qPCR. Each dot represents an animal. p-values we calculated using a two-tailed t-test. **(C)** Schematic representation of the *Rtl1* imprinted locus. The differentially methylated region (DMR) of this locus, which is methylated only in the paternal allele leads to imprinted expression of multiple genes including *Rtl1* and *Rtl1-as* whose expressions overlap in an antisense manner. *Rtl1-as* is a primary miRNA transcript that is processed into 6 distinct miRNAs. **(E)** Identification of AGO2 cleavage products in wild-type (+/+) but not catalytic mutant (D598A/D598A) samples. The identity of the miRNA triggering the cleavage event is shown on the right. Closed and open arrowheads denote mature and star strands respectively. **(D)** Representation of the pairing between miRNAs of the *miR-136~miR-431* cluster (orange) and the *Rtl1* transcript (blue). The position of the predicted cleavage site is indicated, along with the number of times that cleavage site was sequenced.

**Fig. 6. *Rtl1* upregulation causes endothelial cell defects. (A)** Schematic representation of the endothelial cell isolation strategy. **(B, C)** *Rtl1* is expressed and upregulated in endothelial cells isolated from both placentas (B) and embryos (C). N.D., not detected. **(D)** Rtl1 expression in heterozygous control (*+/D598A*; grey) and homozygous mutant (*D598A/D598A*; blue) endothelial cell cultures stably infected with dCas9-KRAB control vector or a vector silencing *Rtl1* expression. **(E)** Permeability assay performed on endothelial cells cultured in the absence of VEGF **(F)** Top, schematic representation of features quantified during the tube formation assay. Bottom, representative bright field image of tubes formed when indicated endothelial lines were cultured in Matrigel. **(G-I)** Number of junctions (G), meshes (H), and nodes (I) per field in indicated cell lines. Each dot represents a technical replicate. P-values were calculated using a two-tailed t-test.

**Sup. Fig. 1. Placentomegaly in *Ago2* catalytic mutants. (A)** Relative placental weights of mutants relative to wild-type littermates at E18.5. **(B)** Relative embryo weights of mutants relative to wild-type littermates at E18.5. **(C)** Relative placental efficiency of mutants relative to wild-type littermates at E18.5. Each dot represents an animal. p-values we calculated using a two-tailed t-test. **(D)** Histone H3 phosphorylation as measured by western blot on protein lysates from whole E14.5, E16.6, and E18.5 placentas. **(E)** Flow cytometry analysis of E16.5 placentas using antibodies against the proliferative marker Ki67 and the endothelial cell marker CD31. Plots show overlay of two biological replicates per genotype, each highlighted with a different color. **(F)** Quantification of (E). Each dot represents a biological replicate. P-values were calculated using a two-tailed t-test. **(G)** Schematic representation of a mid-gestation embryo, highlighting which structures are trophectoderm (blue) versus epiblast (pink) derived. **(H)** Genotyping PCR from 4 embryos (E1-E4), showing examples of a $Ago2^{flx/+}$*;Sox2-Cre*$^{-}$ embryo (E1), a $Ago2^{flx/D598A}$*;Sox2-Cre*$^{-}$ embryo (E2), a $Ago2^{flx/+}$*;Sox2-Cre*$^{+}$ embryo (E3), and a $Ago2^{flx/D598A}$*;Sox2-Cre*$^{+}$ embryo (E4).

**Sup. Fig. 2. Skeletal development in the absence of AGO2 catalytic activity. (A-D)** Representative examples of skeletons from E18.5 wild-type and homozygous mutant animals stained with alcian blue (for cartilage) and alizarin red (for bone). The positions of vertebra 21 (V21) and 27 (V27) which correspond to the first lumbar and first sacral vertebras in both genotypes are highlighted in (B). **(E-J)** Length of the bones highlighted in (C-D) for each genotype. Each dot represents an animal. P-values were calculated using a two-tailed t-test.

**Sup. Fig. 3. Endothelial cell dysregulation in AGO2 catalytic mutant mice. (A)** Enrichment analysis for gene signatures from 18 placental types in genes upregulated in *Ago2* catalytic mutants. **(B)** Immunofluorescence staining of mouse placentas with an antibody against the CD31 endothelial cell marker. **(C)** Quantification of vessel area in wild-type and mutant placentas. p-values we calculated using a two-tailed t-test. **(D)** Representative E18.5 fetuses enveloped by the yolk-sac. Major vessels are highlighted. **(E)** Immunofluorescence staining of yolk-sacs with an antibody against the CD31 endothelial cell marker. Error bar, 100 μm.

**Sup. Fig. 4. Detection of cleavage fragments by a modified 5'RACE protocol. (A)** Schematic representation of the protocol. Briefly, polyadenylated transcripts are enriched using oligo-dT magnetic beads and used in a ligation reaction with a 5'adaptor. Transcripts with a 5'cap (represented by black circle) or a 5'OH group as a consequence of RNA degradation are unable to ligate to the adaptor. RNA fragments generated by AGO2 cleavage on the other hand, have a phosphorylated 5' terminus and can be ligated efficiently. Following adaptor ligation, RNAs are reverse-transcribed with a transcript-specific primer and finally amplified by PCR. Amplification is done with a forward primer that binds to the 5' adaptor sequence and a transcript-specific reverse primer. **(B)** Example trace file obtained from the sequencing of the miR-127 cleavage fragments, with pairing of miRNA and target displayed on the top. Dotted line indicates the predicted position of AGO2-mediated cleavage. **(C)** Example trace files showing expected cleavage fragments for miR-136 and miR-433-5p

**Sup. Fig. 5. *Rtl1* overexpression is the cause of endothelial cell defects in *Ago2* catalytic mutants. (A)** Relative *Rtl1* expression in E14.5 placentas and embryos. All values are normalized to the average of the wild-type placenta. Each dot represents a biological replicate. P-values were calculated using a two-tailed t-test. **(B)** Relative *Rtl1* expression from *Ago2*$^{flx/+}$*;Sox2-Cre*$^{+}$ and *Ago2*$^{flx/D598A}$*;Sox2-Cre*$^{+}$ E18.5 placentas. Each dot represents a biological replicate. P-values were calculated using a two-tailed t-test. **(C)** Immunofluorescent staining of endothelial cell cultures of indicated genotypes infected with a control vector or a vector that knocks-down *Rtl1* using the CRISPRi strategy. **(D)** Percentage of Ki67 positive cells in *Ago2*$^{+/D598A}$ control (grey bars) and *Ago2*$^{D598A/D598A}$ mutant (blue bars) endothelial cultures infected with the indicated constructs. Each dot represents a technical replicate. P-values were calculated using a two-tailed t-test. **(E)** Permeability assay performed on endothelial cells cultured in the presence of VEGF, a growth factor that increases vascular permeability, as a percentage of untreated control samples. **(F)** Average lumen area of tubes formed by the endothelial cells cultured in the presence of Matrigel. Each dot represents an independent replicate experiment. p-values we calculated using a two-tailed t-test.
